# Supplementary material for: Conformational changes in the motor ATPase CpaF facilitate a rotary mechanism of Tad pilus assembly
Source: Nat Commun. 2025 Apr 24;16:3839. doi: 10.1038/s41467-025-59009-5 (PMC12019362; doi:10.1038/s41467-025-59009-5)
Supplement: Supplementary file 1 — Supplementary Information [file 41467_2025_59009_MOESM1_ESM.pdf]

Supplementary Information

Conformational changes in the motor ATPase CpaF facilitate a rotary mechanism of Tad pilus assembly

Ian Y. Yen[1,2], Gregory B. Whitfield[3], John L. Rubinstein[1,2,4], Lori L. Burrows[5*], Yves V. Brun[3*], P. Lynne Howell[1,2*]

[1]Program in Molecular Medicine, Peter Gilgan Center for Research and Learning, The Hospital for Sick Children, Toronto, ON M5G 0A4, Canada.
[2]Department of Biochemistry, University of Toronto, Toronto, ON M5S 1A8, Canada.
[3]Département de Microbiologie, Infectiologie et Immunologie, Université de Montréal, Montréal, Québec H3C 3J7, Canada.
[4]Department of Medical Biophysics, University of Toronto, Toronto, ON M5G 1L7, Canada.
[5]Biochemistry and Biomedical Sciences and the Michael G. DeGroote Centre for Infectious Disease Research, McMaster University, Hamilton, ON L8S 4K1, Canada.

*Co-corresponding authors: Lori L. Burrows (lori.burrows@mcmaster.ca), Yves V. Brun (yves.brun@umontreal.ca), P. Lynne Howell (howell@sickkids.ca)

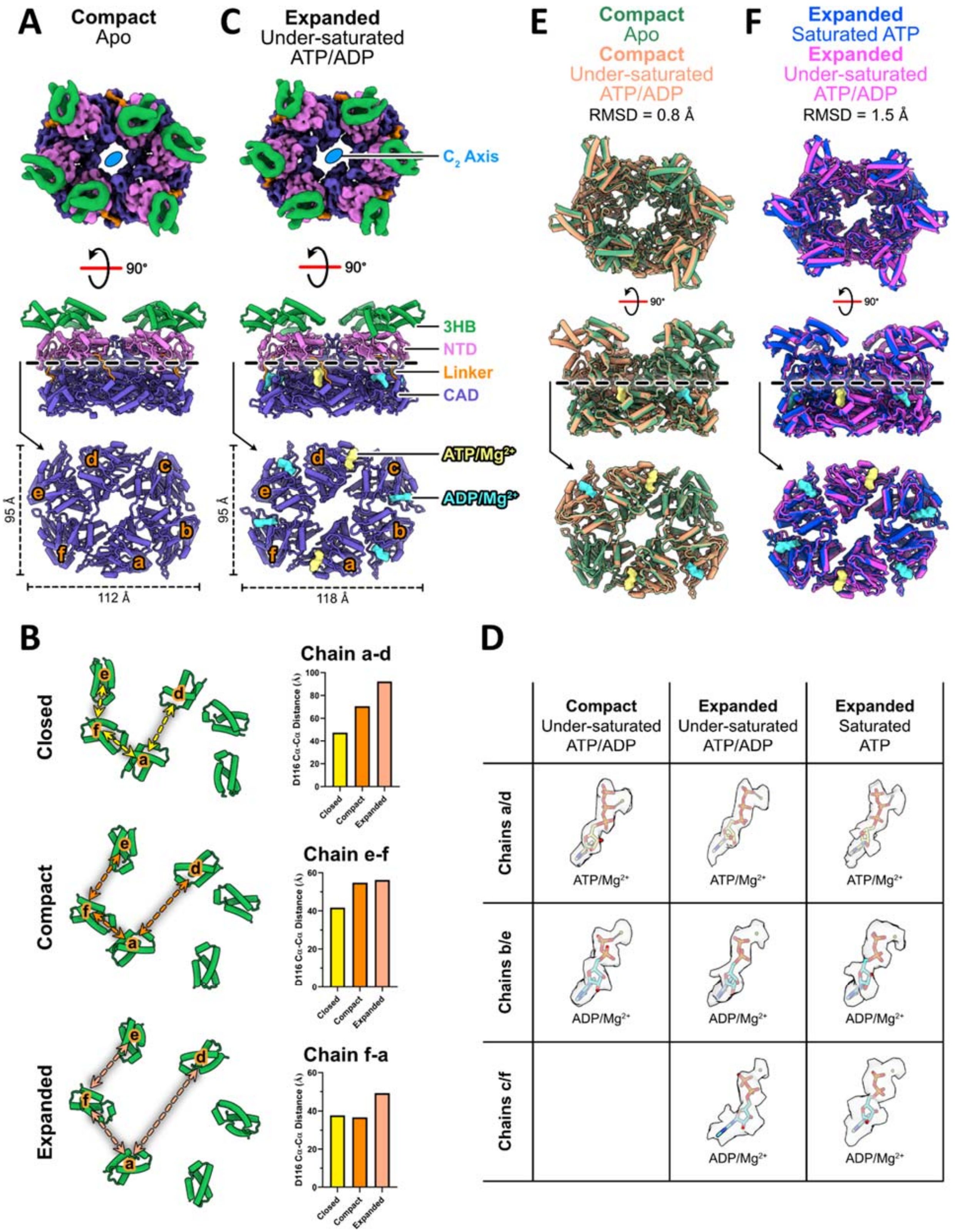

Supplementary Figure 1. Additional CpaF structures, nucleotide densities, structural measurements and alignments. A/C. Additional CpaF structures in the compact and expanded

conformations determined from the apo and under-saturated ATP/ADP datasets, respectively, represented as described in Figure 1. **B.** Cα-Cα distances (Å) of D116 on the 3HB measured for the three CpaF structures. Chain a-d represents the distance across symmetrically positioned 3HBs while chains e-f and f-a represent distances between adjacent 3HBs. Exact measurements are reported in the Source Data file. **D.** Locally sharpened map densities in the active site pockets of all structures harboring nucleotides obtained from the three datasets, and the nucleotides modeled into them. All map densities within each dataset are depicted at the same threshold. **E-F.** The RMSD of the two aligned compact structures from the apo and under-saturated ATP/ADP datasets, and the two expanded structures from the under-saturated ATP/ADP and saturated ATP datasets, was calculated along the entire hexamer.

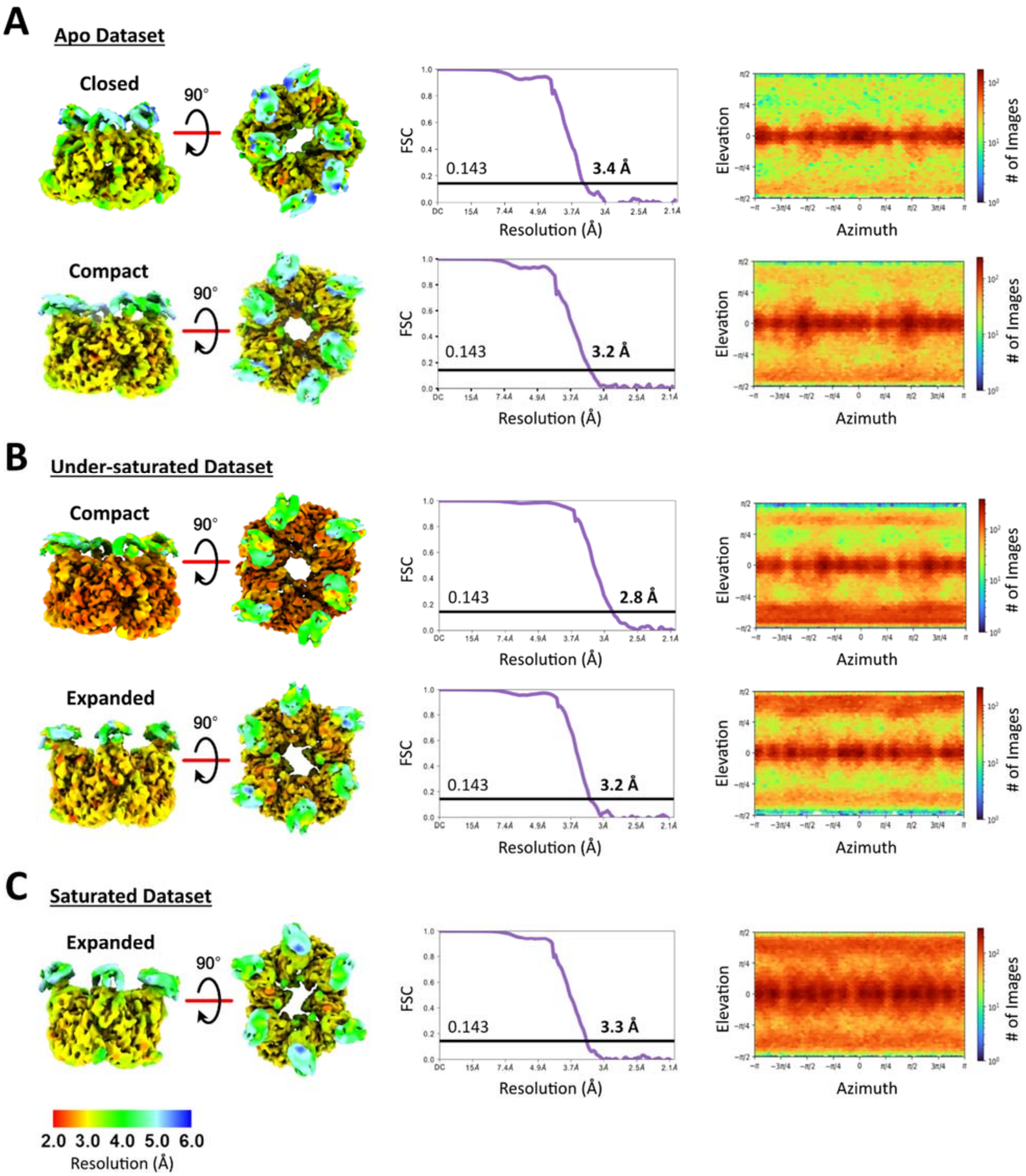

Supplementary Figure 2. Consensus cryo-EM map validation. A-C. (left) Consensus maps of the biological hexamer from the three datasets, depicted in top and side views and colored by local resolution, along with the (middle) corrected Fourier shell correlation (FSC) curves following a gold standard refinement and the (right) orientation distribution plots.

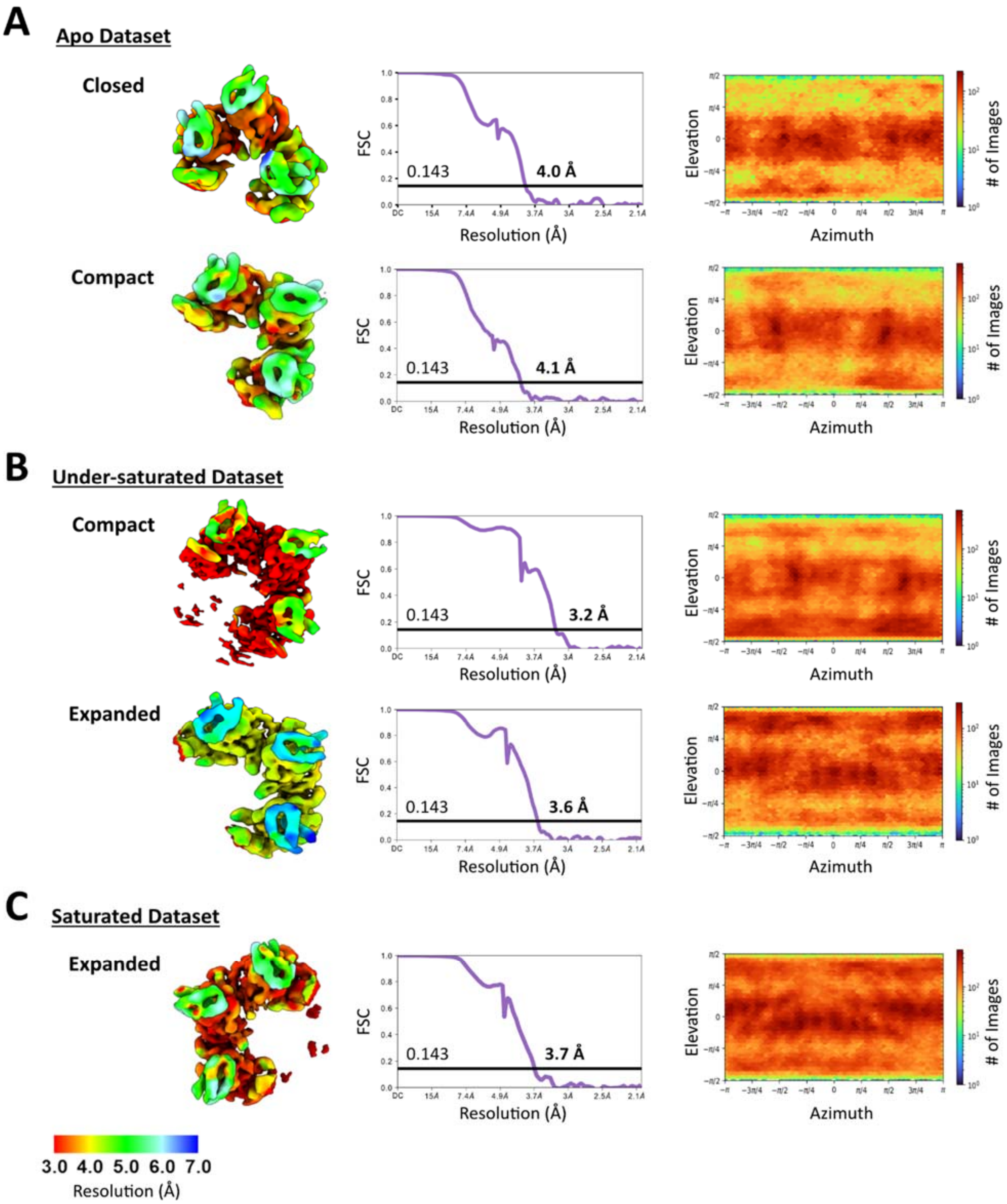

Supplementary Figure 3. Locally refined cryo-EM map validation. A-C. (left) Locally refined maps of the 3HBs and NTDs from the asymmetric trimer in the three datasets, depicted in top view and colored by local resolution, along with the (middle) corrected FSC curves following a gold standard refinement and the (right) orientation distribution plots.

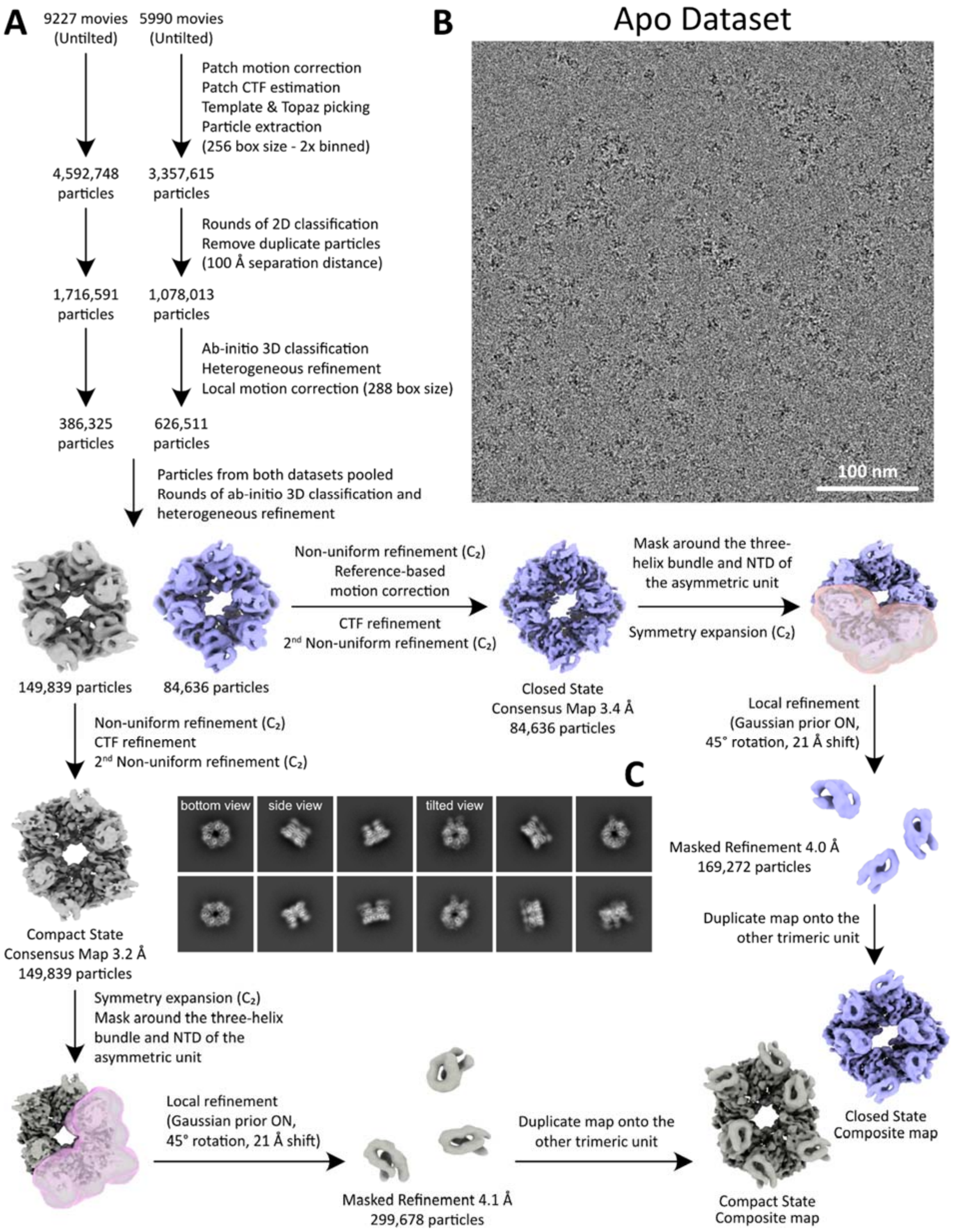

Supplementary Figure 4. Image processing workflow for the apo dataset. A. cryoSPARC processing workflow for obtaining consensus, locally refined, and composite maps of the closed and compact states. **B.** Representative micrograph and **C.** 2D class average images of both states.

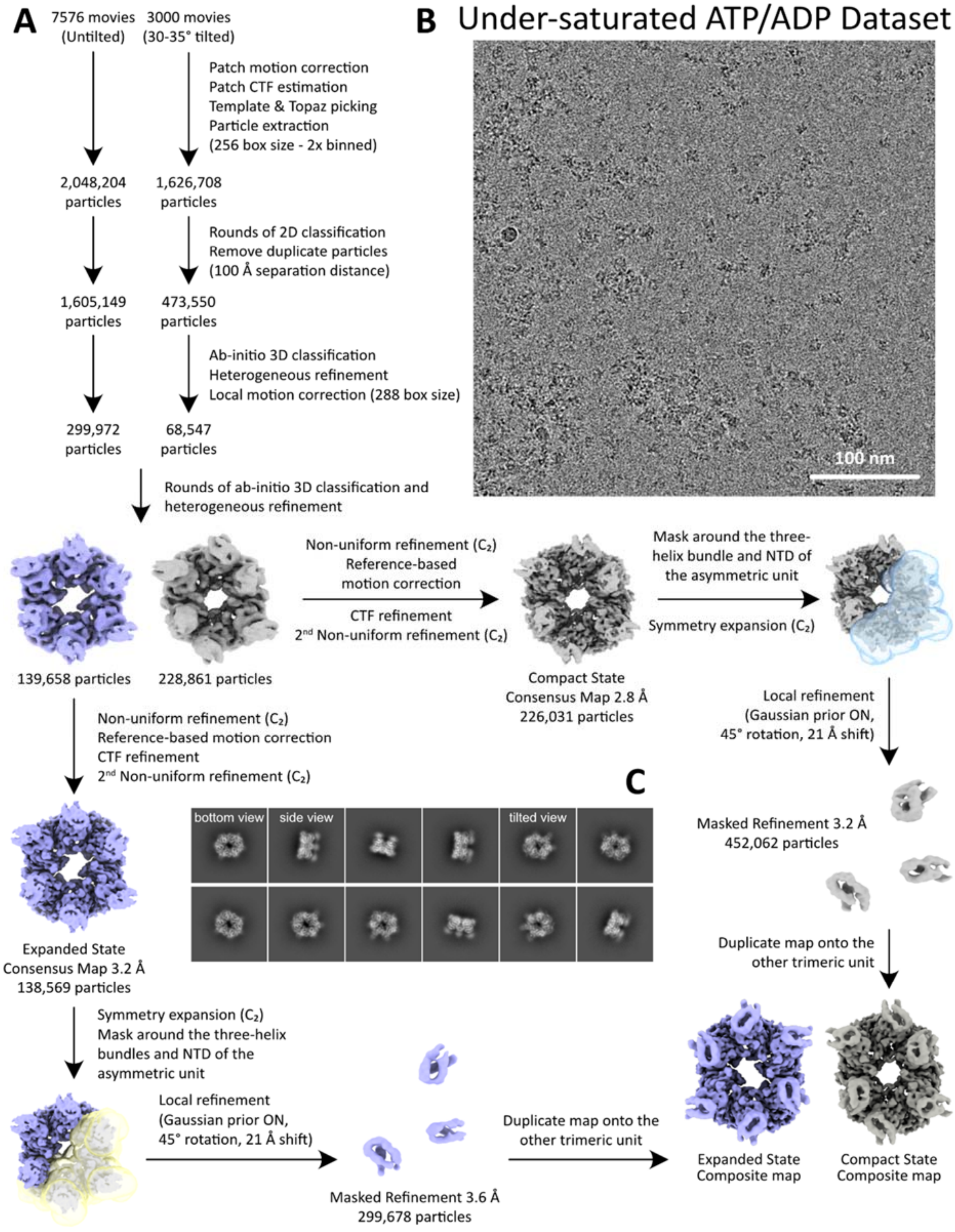

Supplementary Figure 5. Image processing workflow for the under-saturated ATP/ADP dataset. A. cryoSPARC processing workflow for obtaining consensus, locally refined, and composite maps of the compact and expanded states. **B.** Representative micrograph and **C.** 2D class average images of both states.

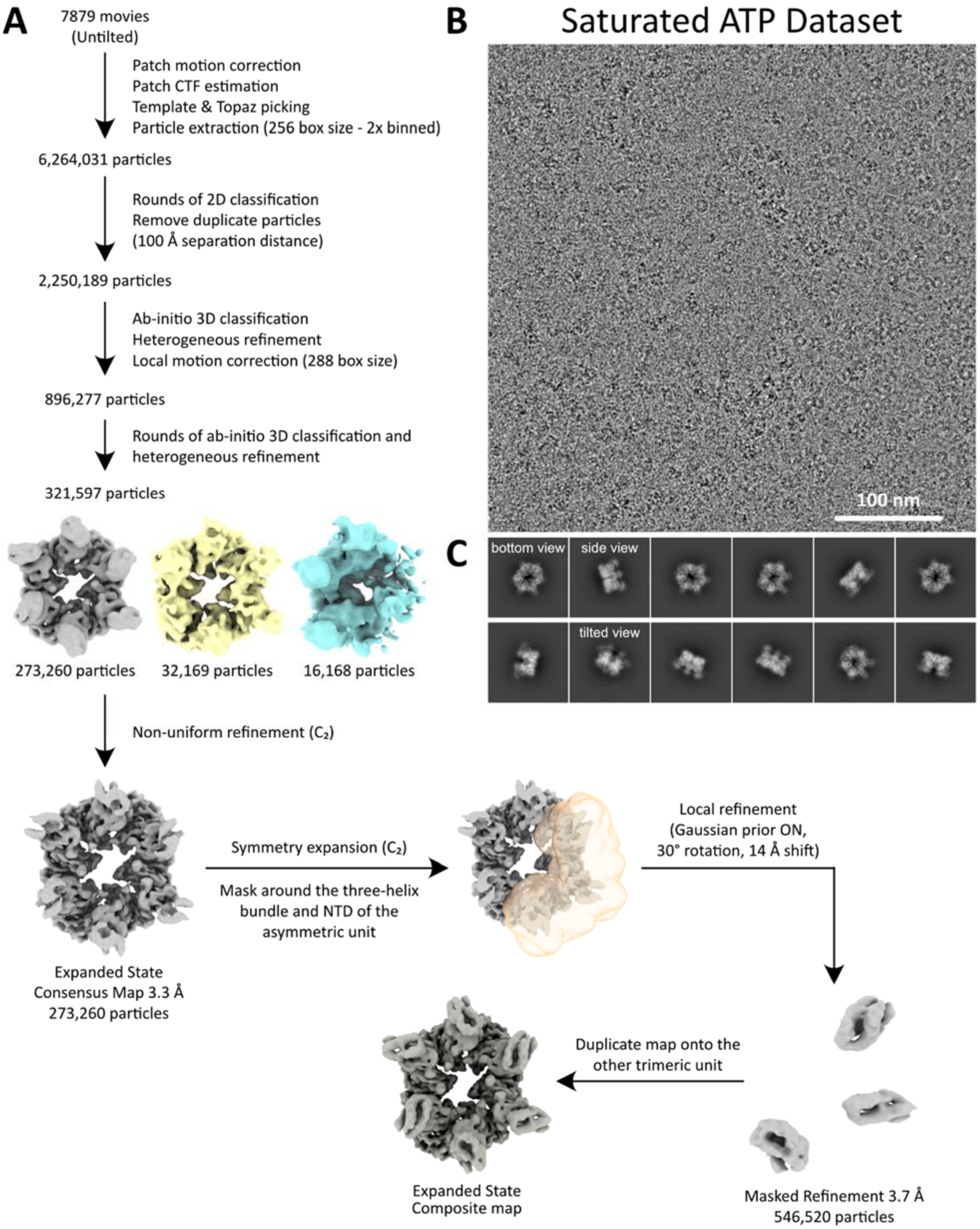

Supplementary Figure 6. Image processing workflow for the saturated ATP dataset. A. cryoSPARC processing workflow for obtaining consensus, locally refined, and composite maps of the expanded state. **B.** Representative micrograph and **C.** 2D class average images.

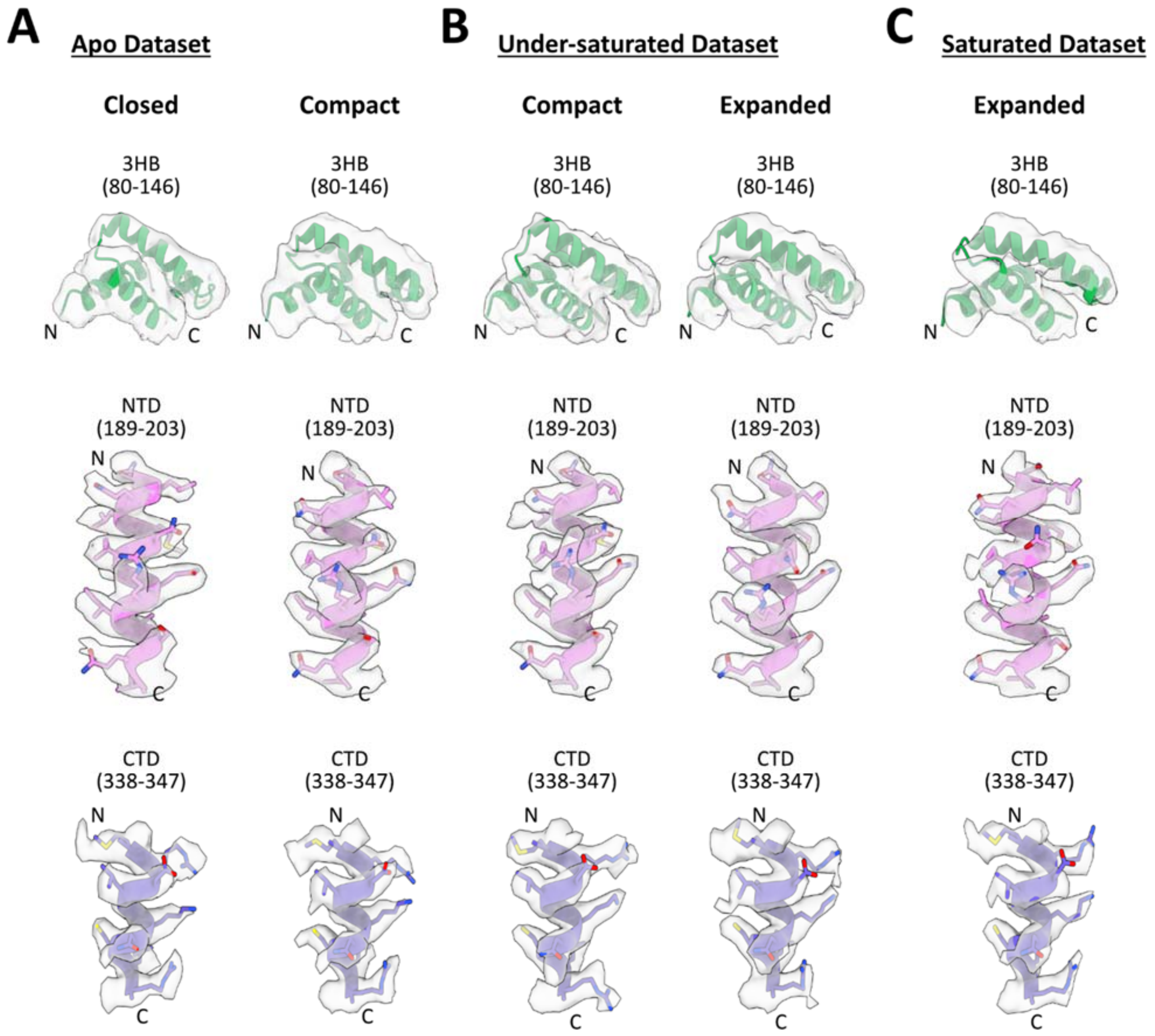

Supplementary Figure 7. Examples of model-in-map fit quality. A-C. (top) Fit of the atomic models into the respective locally refined maps of 3HB between residues D80 and L146. Side chains in this region were not built due to lower map resolution. Fit of the atomic models into the consensus maps of: (middle) the NTD between residues N189 and V203; and (bottom) the CAD between residues M338 and R347.

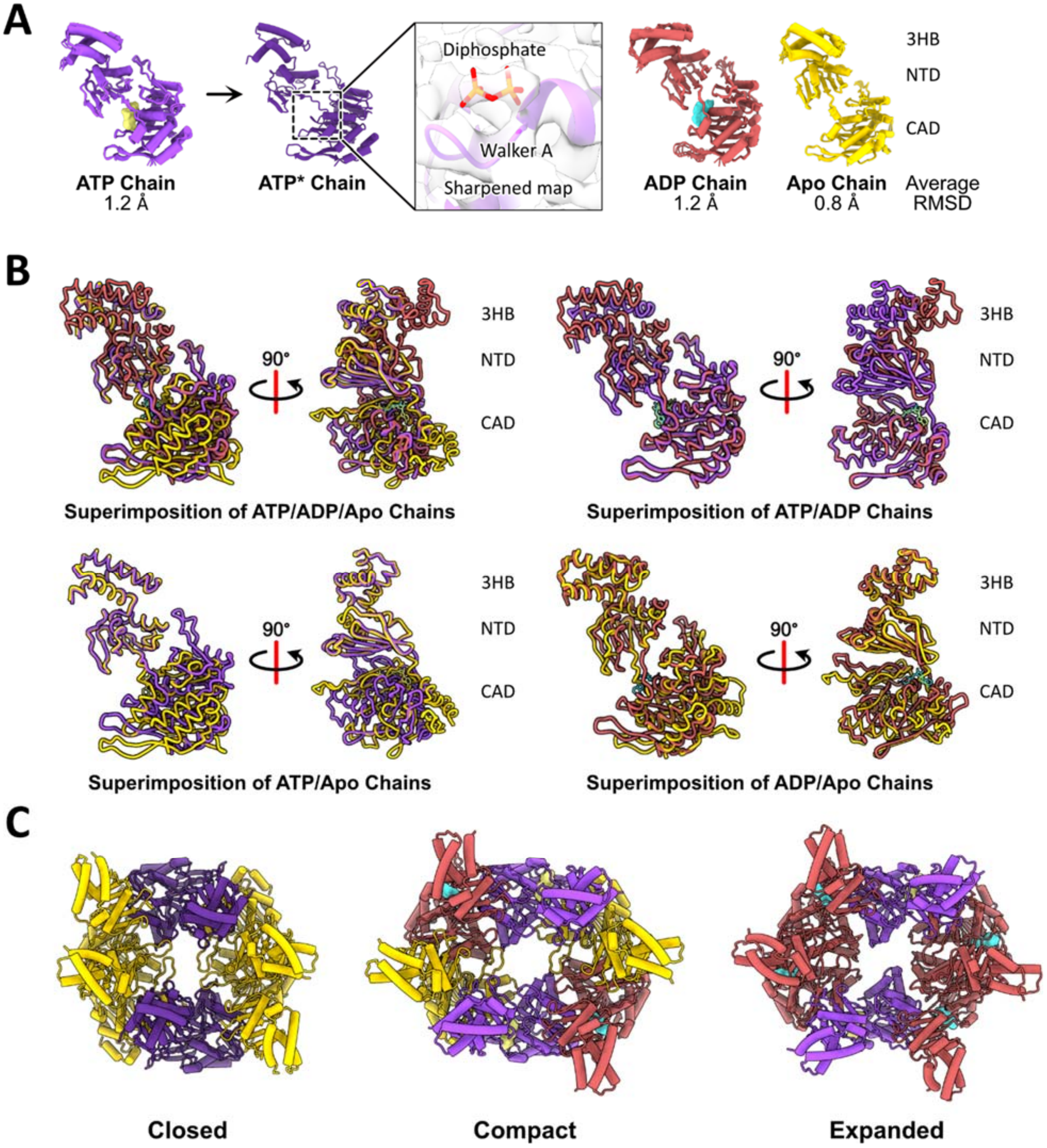

Supplementary Figure 8. Classification and structural alignment of monomeric chains by nucleotide identity. A. Superimposition of Cα atoms from all eighteen chains of the three structures grouped by their bound nucleotide. The average RMSD within each group is shown. Two chains within the ATP group, originating from the closed structure, adopt an ATP-like conformation despite the absence of ATP in the active site, which were designated ATP*. (inset) The residual density present in only the sharpened consensus map of the closed state could be fit with a diphosphate. **B.** Pairwise alignment of the ATP, ADP, and Apo chains from the compact structure, as well as alignment of all three chains, highlighting nucleotide-induced domain

movements. Chains are colored as in panel A. **C.** Atomic models of the closed, compact, and expanded hexamers, colored according to the nucleotide bound in each chain as depicted in panel A.

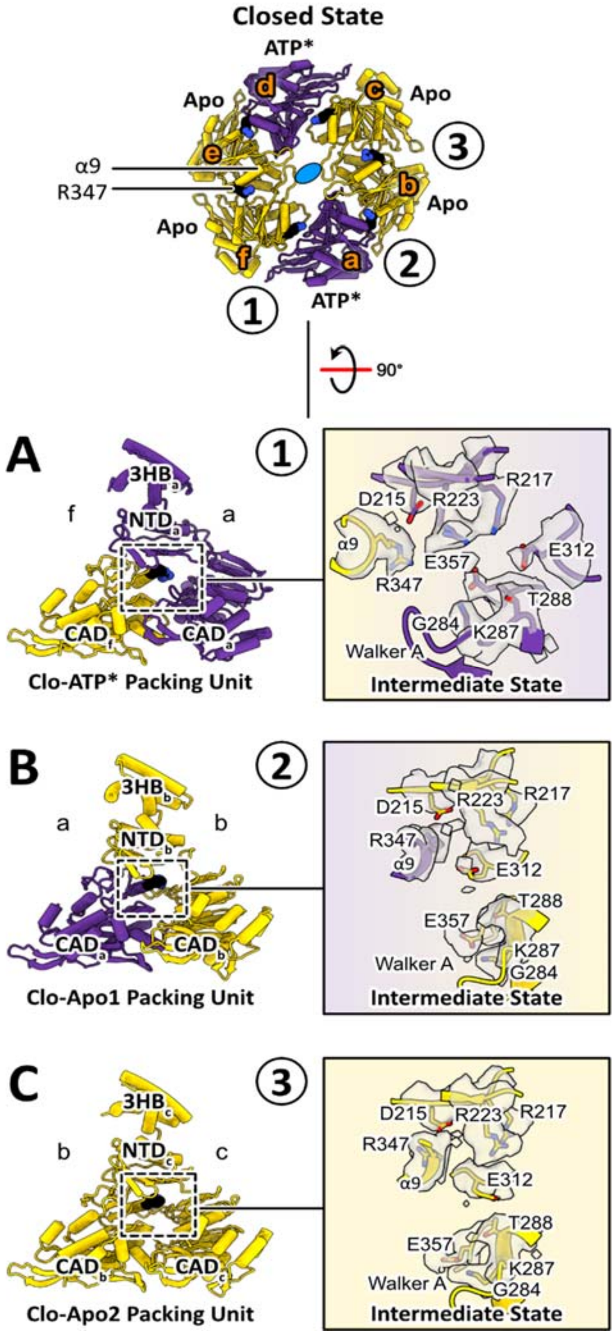

Supplementary Figure 9. Packing unit analysis of the closed structure. A-C. Packing units are numbered 1 to 3 according to their positions within an asymmetric unit of the hexamer. Full chains and their constituent domains are labelled from a to f and colored according to their bound nucleotides – ATP* in dark purple and Apo in gold. The residue R347 on the α9 helix in black is shown as a space-filling model colored by its side chain heteroatom. Active site analysis highlights residues, shown in stick representation, involved in stabilizing bound nucleotides. All residues and nucleotides are encased in their corresponding cryo-EM density (grey for amino acids) contoured at the same threshold level. The side chain rotamers of R217, R223, and R347 are classified as "intermediate".

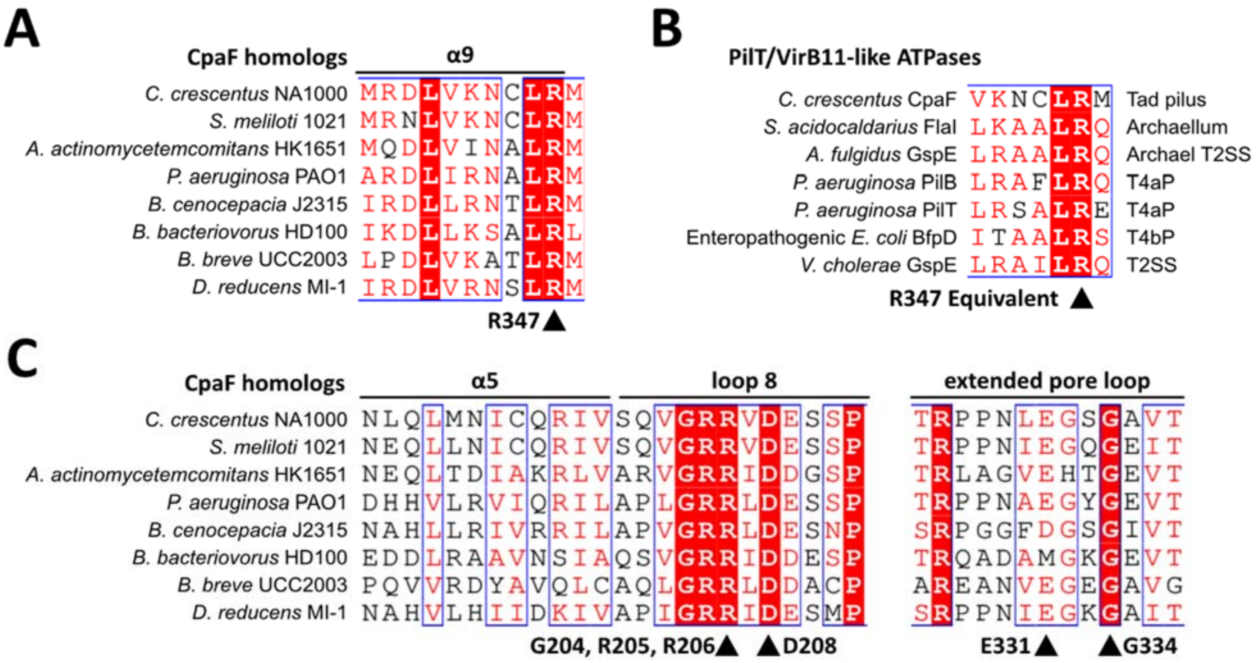

Supplementary Figure 10. Multiple sequence alignments of CpaF. Multiple sequence alignment of **(A)** representative CpaF orthologs from all major bacterial phyla, depicting conserved residue R347 on α9 helix, and (**B)** of PilT/VirB11-like ATPases from all TFF superfamily systems, highlighting conservation of the R347 residue or its equivalent. (**C**) Multiple sequence alignment of representative CpaF orthologs from all major bacterial phyla, highlighting conserved residues in loop 8 (S201-P212) and the extended pore loop (T325-T337).

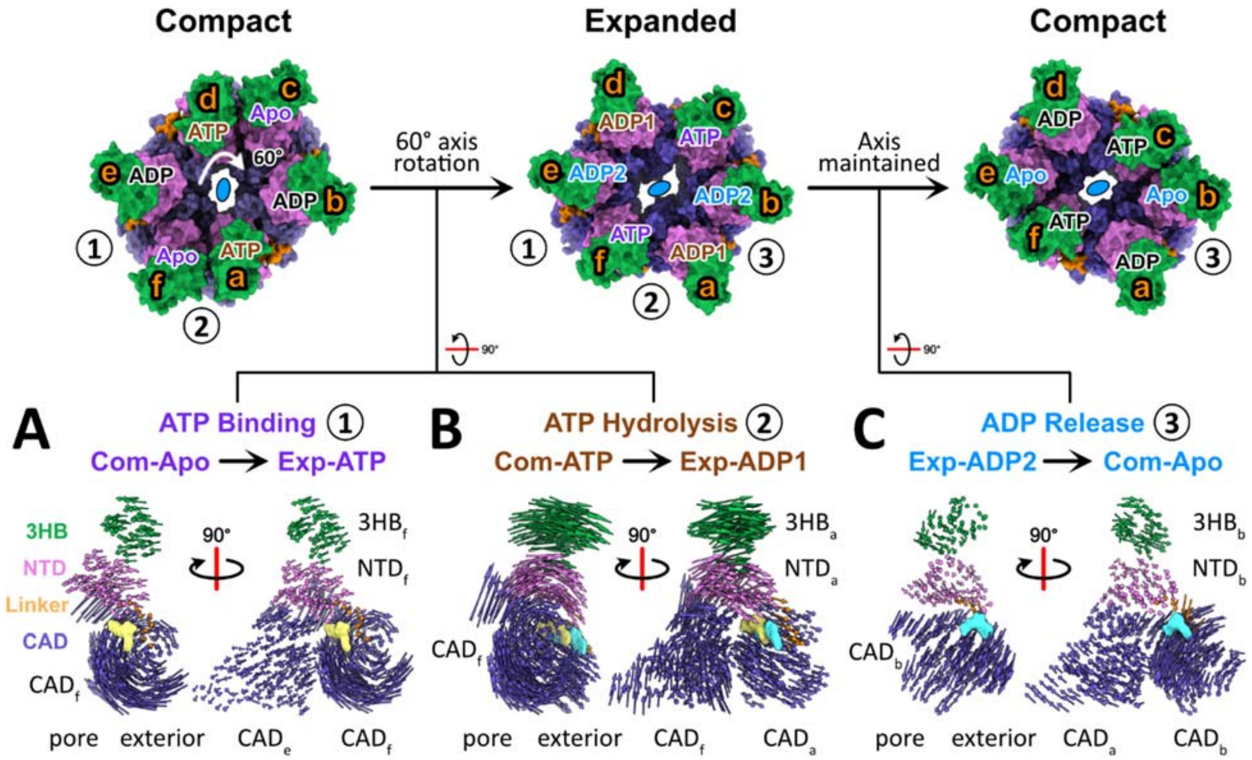

Supplementary Figure 11. Vector diagrams of superimposed packing units. A-C. Pairwise Cα interatomic trajectories from packing unit alignments during each catalytic event. Each arrow represents the distances traveled by a residue. In panels A and C, CAD_e and CAD_a are omitted for clarity.

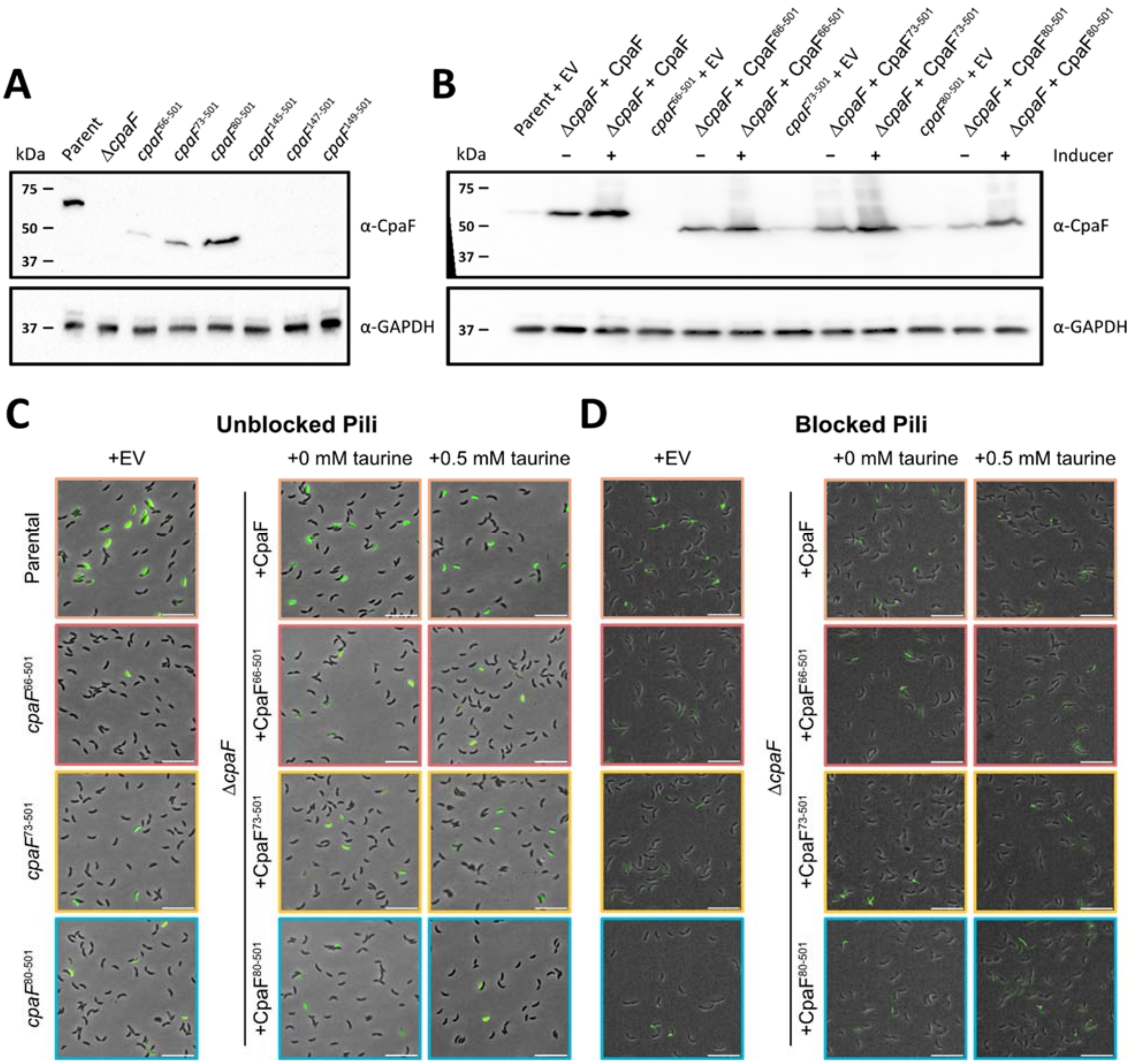

Supplementary Figure 12. Overexpression of CpaF IDR truncation mutants produces similar pilus activity levels. A. Western blot showing expression of CpaF IDR and 3HB truncations from whole cell lysates probed using CpaF-specific antibodies. **B.** Western blot showing expression of CpaF IDR truncation constructs from whole cell lysates with and without addition of 0.5 mM taurine probed using CpaF-specific antibodies. For A and B, antibody against GAPDH was used as a loading control. Source data are provided in the Source Data file **C.** Representative images of a mixed population of *C. crescentus pil*-cys (parental) cells harboring IDR truncations at the native chromosomal locus (left) or expressed from an inducible vector in a Δ*cpaF* background (right), labeled with AF488-maleimide. **D.** Representative images of a mixed population of *C. crescentus pil*-cys (parental) cells harboring IDR truncations at the native chromosomal locus (left) or expressed from an inducible vector in a Δ*cpaF* background, blocked with PEG5000-maleimide and labeled with AF488-maleimide. In panels B-D, cells harboring *cpaF* at the native

chromosomal locus were transformed with empty vector (EV) as a control. Cells in a Δ*cpaF* background were transformed with an inducible vector harboring *cpaF* or truncation variants. Induction of *cpaF* expression was done via addition of 0.5 mM taurine. Scale bars = 10 μm.

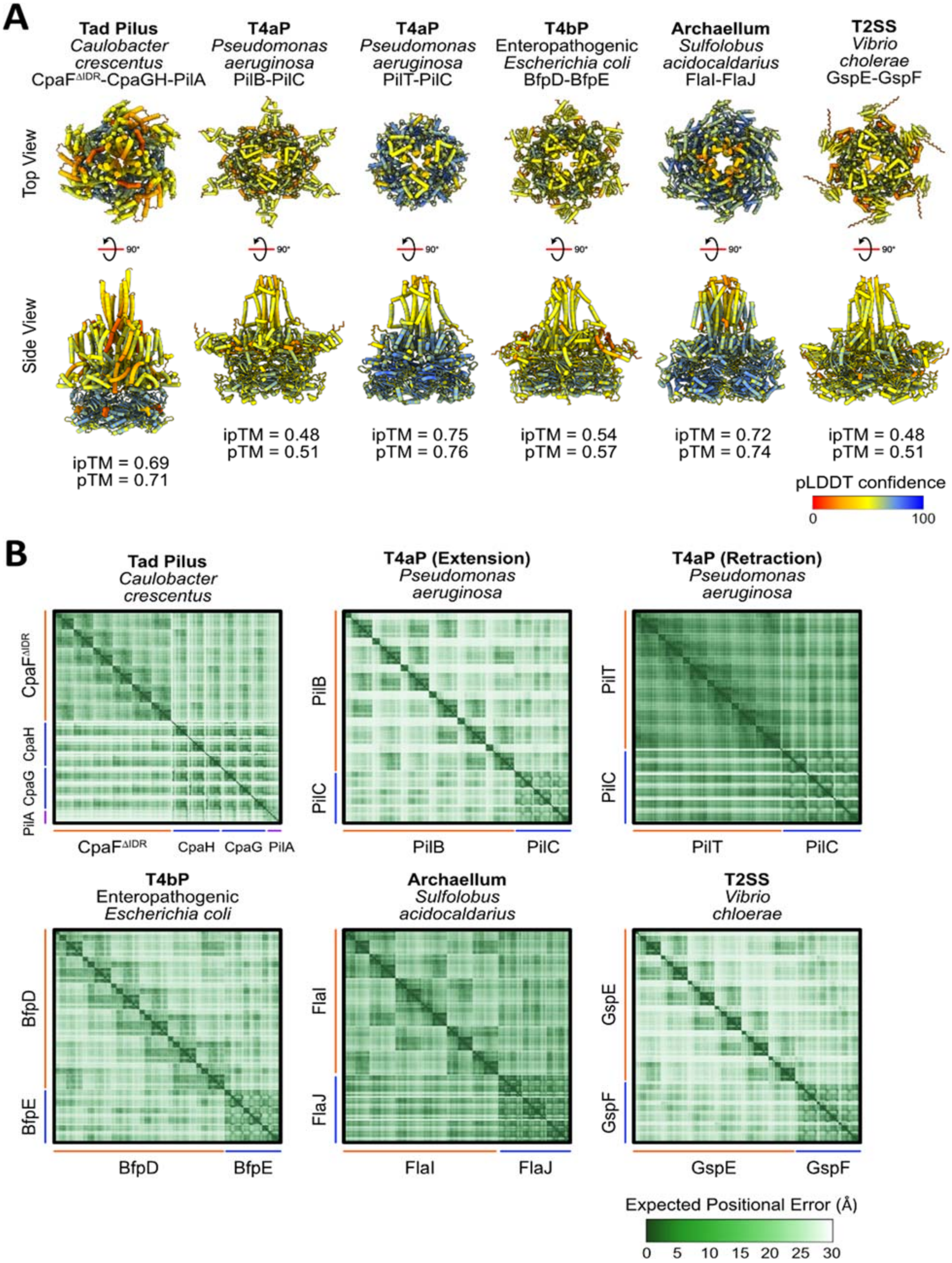
A
Tad Pilus
Caulobacter crescentus
CpaF^ΔIDR-CpaGH-PilA
T4aP
Pseudomonas aeruginosa
PilB-PilC
T4aP
Pseudomonas aeruginosa
PilT-PilC
T4bP
Enteropathogenic Escherichia coli
BfpD-BfpE
Archaellum
Sulfolobus acidocaldarius
FlaI-FlaJ
T2SS
Vibrio cholerae
GspE-GspF
Top View
90°
Side View
ipTM = 0.69
pTM = 0.71
ipTM = 0.48
pTM = 0.51
ipTM = 0.75
pTM = 0.76
ipTM = 0.54
pTM = 0.57
ipTM = 0.72
pTM = 0.74
ipTM = 0.48
pTM = 0.51
pLDDT confidence
0
100
B
Tad Pilus
Caulobacter crescentus
CpaF^ΔIDR
CpaH
CpaG
PilA
T4aP (Extension)
Pseudomonas aeruginosa
PilB
PilC
T4aP (Retraction)
Pseudomonas aeruginosa
PilT
PilC
T4bP
Enteropathogenic Escherichia coli
BfpD
BfpE
Archaellum
Sulfolobus acidocaldarius
FlaI
FlaJ
T2SS
Vibrio chloerae
GspE
GspF
Expected Positional Error (Å)
0 5 10 15 20 25 30

Supplementary Figure 13. AlphaFold3 prediction confidence of TFF superfamily motor subcomplexes. A. Highest-ranking AlphaFold3 predicted models of the motor subcomplexes from the TFF superfamily systems depicted in top and side views and colored by their respective predicted local distance difference test (pLDDT) scores, ranging from 0-100. The interface predicted template modeling (ipTM) and predicted template modeling (pTM) values from each prediction are shown at the bottom. T4aP, type IVa pilus; T4bP, type IVb pilus; T2SS, type II secretion system. **B.** Residue-residue predicted alignment error (PAE) plot for each prediction, which gives a distance error for every residue pair and estimates the positional error at a specific residue when the predicted and true structures are aligned. The values range from 0-30 Å. Corresponding proteins associated with each plot is shown with ATPases in orange, platform proteins in blue, and major pilin in purple.

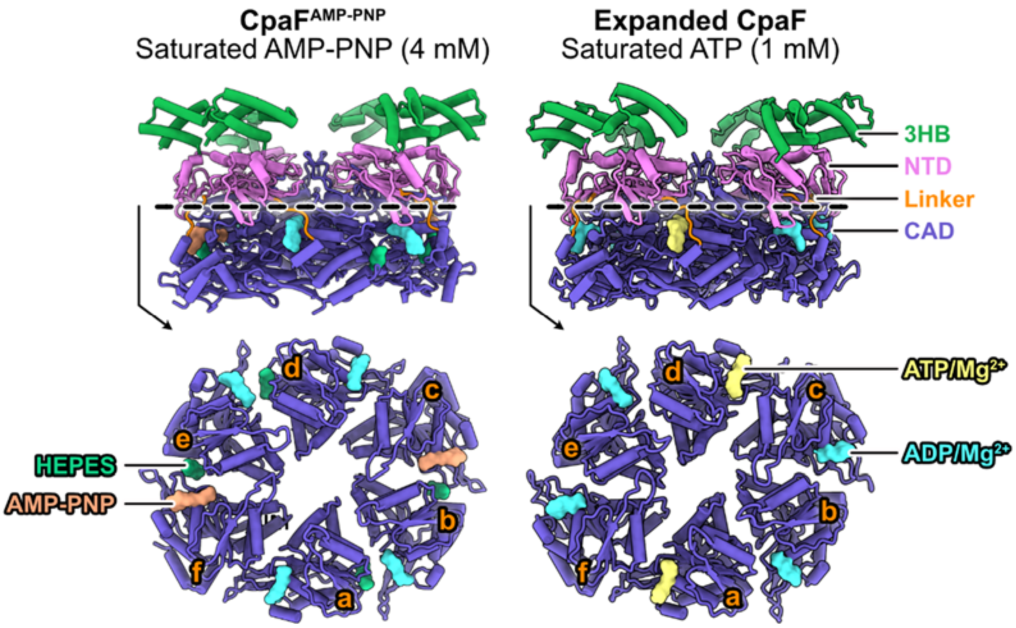

Supplementary Figure 14. Comparison of nucleotide occupancy of two independently determined CpaF structures. (Left) Atomic model of CpaF$_{\text{AMP-PNP}}$ (PDB: 8RKD) shown from the side view and as a cross-section through the NTD (omitted) and CAD (shown). 2 AMP-PNP and 4 ADP molecules occupy the six active sites, while four additional HEPES molecules are bound between chains. (Right) Atomic model of the expanded state (this study) shown in the same orientations as CpaF$_{\text{AMP-PNP}}$. 2 ATP and 4 ADP molecules occupy the six active sites.

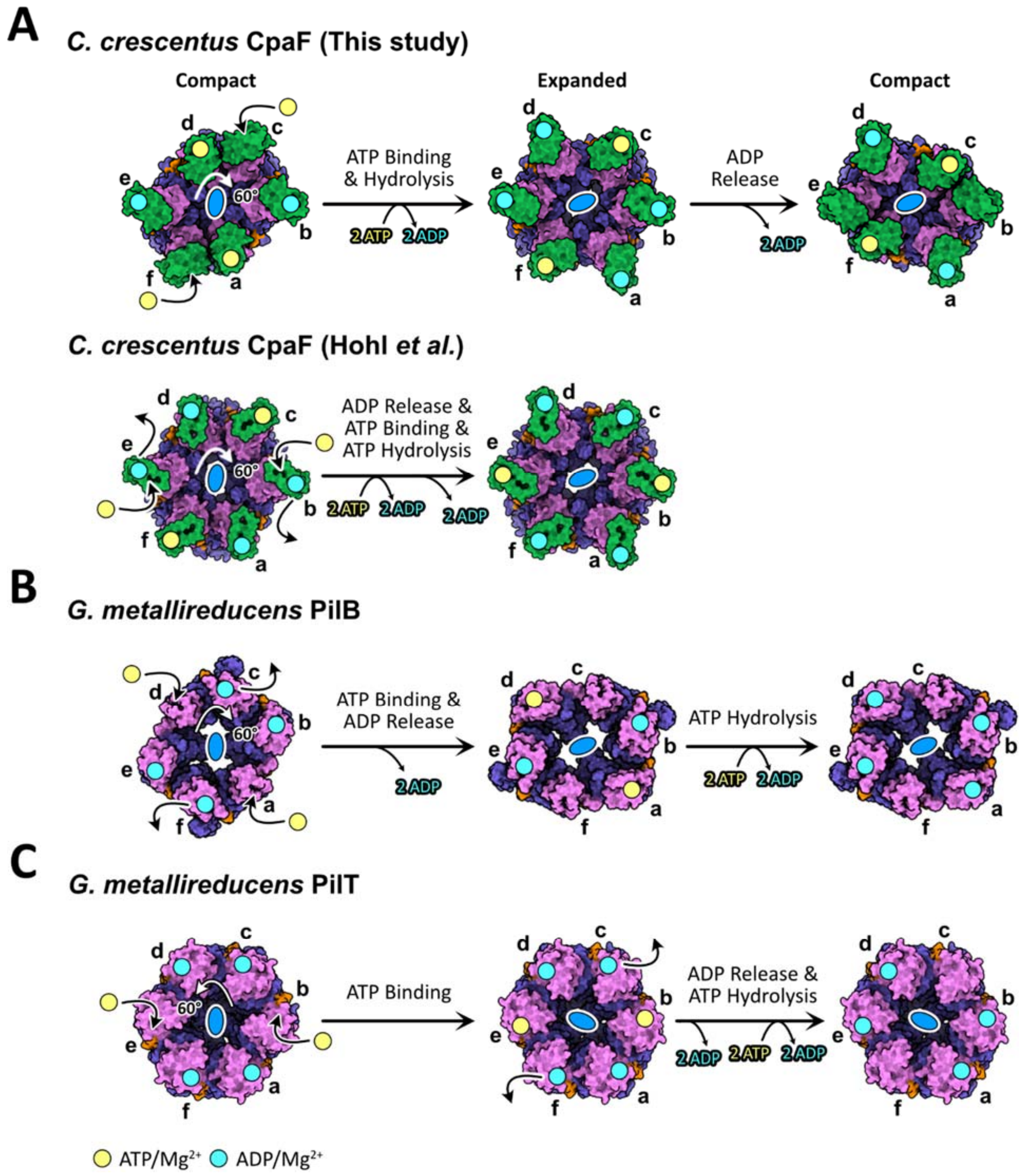

Supplementary Figure 15. Rotary mechanisms of catalysis for CpaF, PilB, and PilT. A. Proposed rotary models of CpaF catalysis. (top) Same model adapted from Figure 8. (bottom) Alternative model[1] in which the expanded state equivalent structure $CpaF_{AMP\text{-}PNP}$ (PDB: 8RKD) undergoes ATP binding, ATP hydrolysis, and ADP release simultaneously to rotate the symmetry axis clockwise by 60°. **B.** Rotary model of *Geobacter metallireducens* PilB[2] (PDB: 5TSH) in which the coupled ATP binding and ADP release rotates the symmetry axis clockwise by 60° while subsequent ATP hydrolysis maintains the axis. **C.** Rotary model of *G. metallireducens* PilT[3] (PDB:

6OKV) in which ATP binding rotates the symmetry axis counterclockwise by 60°, followed by ADP release and ATP hydrolysis events.

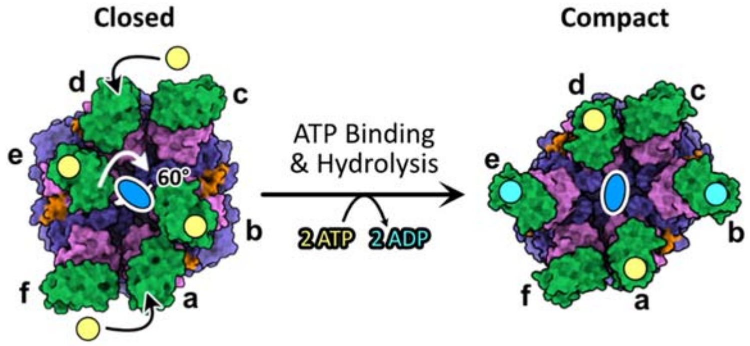

Supplementary Figure 16. Predicted rotary model of the closed-to-compact transition. The closed state may potentially harbor two ATP molecules. ATP binding and hydrolysis rotates the C_2 axis clockwise by 60°, converting CpaF from the closed to the compact state, which can now participate in processive catalysis.

Supplementary Table 1. Cryo-EM collection, refinement, validation statistics

	Closed – Apo<br>(EMD-47431)<br>(PDB 9E24)	Compact – Apo<br>(EMD-47434)<br>(PDB 9E25)	Compact – Under-saturated ATP/ADP<br>(EMD-47437)<br>(PDB 9E26)	Expanded – Under-saturated ATP/ADP<br>(EMD-47440)<br>(PDB 9E27)	Expanded – Saturated ATP<br>(EMD-47444)<br>(PDB 9E29)
**Data Collection and Processing**					
Magnification	75,000x	75,000x	75,000x	75,000x	75,000x
Voltage (kV)	300	300	300	300	300
Electron Exposure (e^-/Å^2)	36.7<br>42	36.7<br>42	36.7	36.7	42
Tilt Angle	0°	0°	0°<br>30°	0°<br>30°	0°
Defocus range (μm)	1.0-2.0	1.0-2.0	1.0-2.0	1.0-2.0	1.0-2.0
Pixel Size (Å)	1.03	1.03	1.03	1.03	1.03
Symmetry Imposed	C2	C2	C2	C2	C2
Initial Particle Images (no.)	1,078,013<br>1,716,591	1,078,013<br>1,716,591	1,605,149<br>473,550	1,605,149<br>473,550	2,250,189
Final Particle Images (no.)	84,636	149,839	226,031	138,569	273,260
Map Resolution (Å)	3.4	3.2	2.8	3.2	3.3
FSC Threshold	0.143	0.143	0.143	0.143	0.143
Map Resolution Range (Å)	2.2-10.1	2.2-9.3	2.2-26.7	2.2-9.6	2.2-9.4
**Refinement**					
Initial Model Used	AlphaFold2	AlphaFold2	AlphaFold2	AlphaFold2	AlphaFold2
Model Resolution (Å)	3.6	3.4	3.0	3.5	3.5
FSC Threshold	0.5	0.5	0.5	0.5	0.5
Sharpening *B* Factor (Å)	Sharpened Locally	Sharpened Locally	Sharpened Locally	Sharpened Locally	Sharpened Locally
Model Composition					
Non-Hydrogen Atoms	35898	35473	36064	36138	36141
Protein Residues	2532	2532	2532	2532	2532
Ligands	0	0	8	12	12
Mean *B* factors (Å^2)					
Protein	147.38	130.62	123.39	159.77	171.68
Ligand	0	0	111.05	151.98	149.59
R.m.s Deviations					
Bond Lengths (Å)	0.003	0.003	0.005	0.003	0.004
Bond Angles (°)	0.496	0.461	0.536	0.524	0.593
Validation					
MolProbity Score	1.48	1.57	1.50	1.75	1.88
Clashscore	6.55	5.24	5.82	8.52	10.21
Poor Rotamers (%)	0.06	0.06	1.07	0.11	0.17
Ramachandran plot					
Favored (%)	97.42	95.87	97.14	95.79	94.96
Allowed (%)	2.58	4.13	2.86	4.21	5.04
Disallowed (%)	0	0	0	0	0

	Locally Refined<br>Closed – Apo<br>(EMD-47432)	Locally Refined<br>Compact – Apo<br>(EMD-47435)	Locally Refined<br>Compact – Under-saturated ATP/ADP<br>(EMD-47438)	Locally Refined<br>Expanded – Under-saturated ATP/ADP<br>(EMD-47441)	Locally Refined<br>Expanded – Saturated ATP<br>(EMD-47445)
**Data Collection and Processing**					
Magnification	75,000x	75,000x	75,000x	75,000x	75,000x
Voltage (kV)	300	300	300	300	300
Electron Exposure (e^-/Å^2)	36.7<br>42	36.7<br>42	36.7	36.7	42
Tilt Angle	0°	0°	0°<br>30°	0°<br>30°	0°
Defocus range (μm)	1.0-2.0	1.0-2.0	1.0-2.0	1.0-2.0	1.0-2.0
Pixel Size (Å)	1.03	1.03	1.03	1.03	1.03
Initial Particle Images (no.)	1,078,013<br>1,716,591	1,078,013<br>1,716,591	1,605,149<br>473,550	1,605,149<br>473,550	2,250,189
Final Particle Images (no.)<br>C2 symmetry expanded	169,272	299,678	452,062	277,138	546,520
Map Resolution (Å)	4.0	4.1	3.2	3.6	3.7
FSC Threshold	0.143	0.143	0.143	0.143	0.143
Map Resolution Range (Å)	2.5-50.2	2.4-46.9	2.2-39.4	2.2-47.4	2.3-41.5

Supplementary Table 2. Bacterial/archaeal species, proteins, and their associated UniProt accession numbers used for AlphaFold3 predictions in Figure 7.

System	Organism	ATPase		Platform	
		Protein	Accession #	Protein	Accession #
T4aP	*Pseudomonas aeruginosa*	PilB	P22608	PilC	P22609
T4aP	*Pseudomonas aeruginosa*	PilT	P24559	PilC	P22609
T4bP	Enteropathogenic *Escherichia coli*	BfpD	B7UTD6	BfpE	B7UTD7
Tad Pilus	*Caulobacter crescentus*	$CpaF^{80-501}$	A0A0H3CDS2	CpaG<br>CpaH	A0A0H3CC65<br>A0A0H3CC31
Archaellum	*Sulfolobus acidocaldarius*	FlaI	Q4J9L0	FlaJ	Q4J9L1
T2SS	*Vibrio cholerae*	GspE	A0A7Z7VKT0	GspF	A0A7Z7VJS7

Supplementary Table 3. Bacterial strains, plasmids, and primers used in this study.

Strain	Description	Source
***Escherichia coli* strains**		
DH5α	Cloning strain; F^{-}*mcrA* Δ(*mrr-hsd*RMS-*mcr*BC) φ80*lacZ*ΔM15 Δ*lac*X74 *rec*A1 *ara*D139 Δ(*ara-leu*)7697 *gal*U *gal*K λ–*rps*L(Str^{R}) *end*A1 *nup*G	Invitrogen
Rosetta 2 (DE3)	Protein expression strain; F^{-} *ompT hsdS*$_{B}$(r$_{B}^{-}$ m$_{B}^{-}$) *gal dcm* (DE3) pRARE2 (Cam^{R})	Stratagene
NEB5α	DH5α derivative, *fhuA2Δ*(*argF-lacZ*)*U169 phoA glnV44 Φ80Δ*(*lacZ*)*M15 gyrA96 recA1 relA1 endA1 thi-1 hsdR17*	New England Biolabs
YB8445	NEB5α pNPTS138::Δ*cpaF*	This study
YB10220	NEB5α pNPTS138::*cpaF*$^{66\text{-}501}$	This study
YB10221	NEB5α pNPTS138::*cpaF*$^{73\text{-}501}$	This study
YB10222	NEB5α pNPTS138::*cpaF*$^{80\text{-}501}$	This study
YB10223	NEB5α pNPTS138::*cpaF*$^{145\text{-}501}$	This study
YB10224	NEB5α pNPTS138::*cpaF*$^{147\text{-}501}$	This study
YB10225	NEB5α pNPTS138::*cpaF*$^{149\text{-}501}$	This study
YB10226	NEB5α pJC585^{-}	This study
YB10227	NEB5α pJC585::*cpaF*	This study
YB10228	NEB5α pJC585::*cpaF*$^{66\text{-}501}$	This study
YB10229	NEB5α pJC585::*cpaF*$^{73\text{-}501}$	This study
YB10230	NEB5α pJC585::*cpaF*$^{80\text{-}501}$	This study
***Caulobacter crescentus* strains**		
NA1000	Synchronizable *C. crescentus* lab adapted strain that does not produce holdfast	[4]
YB8288	NA1000 *pilA*^{T36C}, pili can be labelled with maleimide-conjugated fluorophores	[5]
YB8446	NA1000 *pilA*^{T36C} Δ*cpaF*, allelic exchange with plasmid from YB8445 electroporated into YB8288	This study
YB10231	NA1000 *pilA*^{T36C} *cpaF*$^{66\text{-}501}$, allelic exchange with plasmid from YB10220 electroporated into YB8288	This study
YB10232	NA1000 *pilA*^{T36C} *cpaF*$^{73\text{-}501}$, allelic exchange with plasmid from YB10221 electroporated into YB8288	This study
YB10233	NA1000 *pilA*^{T36C} *cpaF*$^{80\text{-}501}$, allelic exchange with plasmid from YB10222 electroporated into YB8288	This study
YB10234	NA1000 *pilA*^{T36C} *cpaF*$^{145\text{-}501}$, allelic exchange with plasmid from YB10223 electroporated into YB8288	This study
YB10235	NA1000 *pilA*^{T36C} *cpaF*$^{147\text{-}501}$, allelic exchange with plasmid from YB10224 electroporated into YB8288	This study
YB10236	NA1000 *pilA*^{T36C} *cpaF*$^{149\text{-}501}$, allelic exchange with plasmid from YB10225 electroporated into YB8288	This study

YB10237	NA1000 *pilA*^{T36C} pJC585^{-}, electroporation of plasmid from YB10226 into YB8288	This study
YB10238	NA1000 *pilA*^{T36C} *cpaF*$^{66-501}$ pJC585^{-}, electroporation of plasmid from YB10226 into YB10231	This study
YB10239	NA1000 *pilA*^{T36C} *cpaF*$^{73-501}$ pJC585^{-}, electroporation of plasmid from YB10226 into YB10232	This study
YB10240	NA1000 *pilA*^{T36C} *cpaF*$^{80-501}$ pJC585^{-}, electroporation of plasmid from YB10226 into YB10233	This study
YB10241	NA1000 *pilA*^{T36C} Δ*cpaF* pJC585::*cpaF*, electroporation of plasmid from YB10227 into YB8466	This study
YB10242	NA1000 *pilA*^{T36C} Δ*cpaF* pJC585::*cpaF*$^{66-501}$, electroporation of plasmid from YB10228 into YB8466	This study
YB10243	NA1000 *pilA*^{T36C} Δ*cpaF* pJC585::*cpaF*$^{73-501}$, electroporation of plasmid from YB10229 into YB8466	This study
YB10244	NA1000 *pilA*^{T36C} Δ*cpaF* pJC585::*cpaF*$^{80-501}$, electroporation of plasmid from YB10230 into YB8466	This study
**Plasmid**		
pNPTS138	Litmus 38 derivative, *nptI oriT sacB, Kan*^{R}; used for allelic exchange in *C. crescentus*	M.R.K Alley, unpublished
pNPTS138::Δ*cpaF*	pNPTS138 containing 646 bp upstream of *cpaF* codon 17 fused to 637 bp downstream of *cpaF* codon 486 at the EcoRV site; used to generate an in-frame, markerless deletion of the *cpaF* ORF from the NA1000 genome	This study
pNPTS138::*cpaF*$^{66-501}$	pNPTS138 containing 477 bp upstream of and including the *cpaF* start codon fused to 514 bp downstream of *cpaF* codon 65 at the EcoRV site; used to remove codons 2-65 of the *cpaF* ORF from the NA1000 genome	This study
pNPTS138::*cpaF*$^{73-501}$	pNPTS138 containing 477 bp upstream of and including the *cpaF* start codon fused to 496 bp downstream of *cpaF* codon 72 at the EcoRV site; used to remove codons 2-72 of the *cpaF* ORF from the NA1000 genome	This study
pNPTS138::*cpaF*$^{80-501}$	pNPTS138 containing 477 bp upstream of and including the *cpaF* start codon fused to 472 bp downstream of *cpaF* codon 79 at the EcoRV site; used to remove codons 2-79 of the *cpaF* ORF from the NA1000 genome	This study
pNPTS138::*cpaF*$^{145-501}$	pNPTS138 containing 477 bp upstream of and including the *cpaF* start codon fused to 506 bp downstream of *cpaF* codon 144 at the EcoRV site; used to remove codons 2-144 of the *cpaF* ORF from the NA1000 genome	This study
pNPTS138::*cpaF*$^{147-501}$	pNPTS138 containing 477 bp upstream of and including the *cpaF* start codon fused to 500 bp downstream of *cpaF* codon 146 at the EcoRV site; used to remove codons 2-146 of the *cpaF* ORF from the NA1000 genome	This study

pNPTS138::*cpaF*$^{149-501}$	pNPTS138 containing 477 bp upstream of and including the *cpaF* start codon fused to 494 bp downstream of *cpaF* codon 148 at the EcoRV site; used to remove codons 2-148 of the *cpaF* ORF from the NA1000 genome	This study
pJC585	*nptII tauR Ptau-rfp, Kan*^{R}; RFP reporter under the control of a taurine inducible promoter	J. Chen, unpublished
pJC585^{-}	pJC585 digested with KpnI to remove part of the vector-encoded rfp sequence, used as an empty vector control	This study
pJC585::*cpaF*	pJC585 containing *cpaF* fused to a synthetic RBS, inserted between the EcoRI and BamHI sites, under the control of a taurine-inducible promoter	This study
pJC585::*cpaF*$^{66-501}$	pJC585 containing *cpaF*$^{66-501}$ fused to a synthetic RBS, inserted between the EcoRI and BamHI sites, under the control of a taurine-inducible promoter	This study
pJC585::*cpaF*$^{73-501}$	pJC585 containing *cpaF*$^{73-501}$ fused to a synthetic RBS, inserted between the EcoRI and BamHI sites, under the control of a taurine-inducible promoter	This study
pJC585::*cpaF*$^{80-501}$	pJC585 containing *cpaF*$^{80-501}$ fused to a synthetic RBS, inserted between the EcoRI and BamHI sites, under the control of a taurine-inducible promoter	This study
pET28a	IPTG-inducible expression vector encoding N-terminal hexa-histidine tag, a thrombin cleavage site, and an optional C-terminal hexahistidine tag, Kan^{R}	Novagen
pET28a::CpaF	pET28a with WT *C. crescentus* NA1000 *cpaF* fused to an N-terminal hexa-histidine tag; Kan^{R}	This study
pET28a::CpaF$^{66-501}$	pET28a::CpaF corresponding to residues 66-501	This study
pET28a::CpaF$^{73-501}$	pET28a::CpaF corresponding to residues 73-501	
pET28a::CpaF$^{80-501}$	pET28a::CpaF corresponding to residues 80-501	This study
pET28a::CpaF^{K287A}	pET28a::CpaF with a point mutation K287A	This study
**Primer**		
pET28cpaF-FI	**GCGCGGCAGCCAT**ATGTTCGGAAAGCGCGACTCGTCAGC	This study
pET28cpaF-RI	**CTTGTCGACGGAGCTCGAATTC**CTACTCCGCCGCGTCGAGGGCTT	This study
pET28cpaF-FV	AAGCCCTCGACGCGGCGGAGTAG**GAATTCGAGCTCCGTCGACAAG**	This study
pET28cpaF-RV	GCTGACGAGTCGCGCTTTCCGAACAT**CATATGGCTGCCGCGC**	This study
pET28cpaF66-FI	**GCCGCGCGGCAGCCAT**CAGGGCCAGCCGCAAACGG	This study
pET28cpaF66-RI	**CCGCAAGCTTGTCGACGGAGCTCGAATTC**CTACTCCGCCGCGTCGAGGGCTT	This study
pET28cpaF66-FV		This study

	**AAGCCCTCGACGCGGCGGAGTAG**GAATTCGAGC	
pET28cpaF66-RV	TCCGTCGACAAGCTTGCGG	This study
pET28cpaF73-FI	CCGTTTGCGGCTGGCCCTG**ATGGCTGCCGCGCGG C**	This study
	**GCCGCGCGGCAGCCAT**AACATCGTCCGTGAGCAG AGCGACTAC	
pET28cpaF73-RI	**CCGCAAGCTTGTCGACGGAGCTCGAATTC**CTACT CCGCCGCGTCGAGGGCTT	This study
pET28cpaF73-FV	AAGCCCTCGACGCGGCGGAGTAG**GAATTCGAGCT CCGTCGACAAGCTTGCGG**	This study
pET28cpaF73-RV	GTAGTCGCTCTGCTCACGGACGATGTT**ATGGCTGC CGCGCGGC**	This study
pET28cpaF80-FI	**GCCGCGCGGCAGCCAT**GACTACTACCACGCCACC AAGACCACGATCTTC	This study
pET28cpaF80-RI	**CCGCAAGCTTGTCGACGGAGCTCGAATTC**CTACT CCGCCGCGTCGAGGGCTT	This study
pET28cpaF80-FV	**AAGCCCTCGACGCGGCGGAGTAG**GAATTCGAGC TCCGTCGACAAGCTTGCGG	This study
pET28cpaF80-RV	**GAAGATCGTGGTCTTGGTGGCGTGGTAGTAGTC** ATGGCTGCCGCGCGGC	This study
pET28acpaFK287A -FI	GGGTTCGGGCGCGACGACGCTGC	This study
pET28acpaFK287A -RI	GCAGCGTCGTCGCGCCCGAACCC	This study
Δ*cpaF*-upF	**AATTCTGGAT**CCAGCCTGACCGCCTG	This study
Δ*cpaF*-upR	*AGCCCGTA*CGGCGCCTTGGGATCGCC	This study
Δ*cpaF*-downF	*AGGCGCCG*TACGGGCTGGAGCGCGAGC	This study
Δ*cpaF*-downR	**AAGCTTCCTGCAG**TCTCGGTCAGGAT	This study
cpaF-trunc-upF	**GCCAAGCTTCTCTGCAGGAT**TTCGAGGAAGTGAC CCAGAAGATCC	This study
cpaF-trunc-66-upR	*CCGTTTGCGGCTGGCCCTG*CATCTACTTCTTCTTGAA CAGGCCCGAG	This study
cpaF-trunc-66-downF	CAGGGCCAGCCGCAAACGG	This study
cpaF-trunc-73-upR	*TAGTCGCTCTGCTCACGGACGATGTT*CATCTACTTCTT CTTGAACAGGCCCGAG	This study
cpaF-trunc-73-downF	AACATCGTCCGTGAGCAGAGCGACTA	This study
cpaF-trunc-80-upR	*GGTCTTGGTGGCGTGGTAGTAGTC*CATCTACTTCTTC TTGAACAGGCCCGAG	This study
cpaF-trunc-80-downF	GACTACTACCACGCCACCAAGACC	This study

cpaF-trunc-145-upR	*AGCGGGCCATAGCCGAGGAC*CATCTACTTCTTCTTGAACAGGCCCGAG	This study
cpaF-trunc-145-downF	GTCCTCGGCTATGGCCCGCT	This study
cpaF-trunc-147-upR	*GCTCCAGCGGGCCATAGCC*CATCTACTTCTTCTTGAACAGGCCCGAG	This study
cpaF-trunc-147-downF	GGCTATGGCCCGCTGGAGC	This study
cpaF-trunc-149-upR	*CAGCGGCTCCAGCGGGCC*CATCTACTTCTTCTTGAACAGGCCCGAG	This study
cpaF-trunc-149-downF	GGCCCGCTGGAGCCGCTG	This study
cpaF-trunc-IDR-downR	**GCGAATTCGTGGATCCAGAT**TCGGACCATCCAGCGCCAAGG	This study
cpaF-trunc-3HB-downR	**GCGAATTCGTGGATCCAGAT**AGTTCGGCGGCGTCCTCGCA	This study
cpaF-comp-F	GTGTG**GAATTC*TTTAAGAAGGAGATATACAT***ATGTTCGGAAAGCGCGACTCGTCAG	This study
cpaF-comp-66-F	GAGTG**GAATTC*TTTAAGAAGGAGATATACAT***ATGCAGGGCCAGCCGCAAACGG	This study
cpaF-comp-73-F	GTGT**GGAA*TTCTTTAAGAAGGAGATATACAT***ATGAACATCGTCCGTGAGCAGAGCGACTA	This study
cpaF-comp-79-F	GTGT**GGAA*TTCTTTAAGAAGGAGATATACAT***ATGGACTACTACCACGCCACCAAGACC	This study
cpaF-comp-R	GTGT**GGGATCC**CTACTCCGCCGCGTCGAGG	This study

*Restriction sites and sequences for Gibson assembly into destination plasmids are bolded; regions of complementarity to the target amplicon are underlined; regions of reverse complementarity (to facilitate splicing) are italicized; synthetic ribosomal binding sites are in bold italics; point mutations are indicated in red.

SUPPLEMENTARY REFERENCES

1. Hohl, M., Banks, E. J., Manley, M. P., Le, T. B. K. & Low, H. H. Bidirectional pilus processing in the Tad pilus system motor CpaF. *Nat Commun* **15**, 6635 (2024).

2. McCallum, M., Tammam, S., Khan, A., Burrows, L. L. & Howell, P. L. The molecular mechanism of the type IVa pilus motors. *Nat Commun* **8**, (2017).

3. McCallum, M. *et al.* Multiple conformations facilitate PilT function in the type IV pilus. *Nat Commun* **10**, (2019).

4. Evinger, M. & Agabian, N. Envelope-Associated Nucleoid from *Caulobacter crescentus* Stalked and Swarmer Cells. *J Bacteriol* **132**, 294–301 (1977).

5. Ellison, C. K. *et al.* Obstruction of pilus retraction stimulates bacterial surface sensing. *Science (1979)* **358**, 535–538 (2017).
