# Supplementary material for: Conformational changes in the motor ATPase CpaF facilitate a rotary mechanism of Tad pilus assembly
Source: Nat Commun. 2025 Apr 24;16:3839. doi: 10.1038/s41467-025-59009-5 (PMC12019362; doi:10.1038/s41467-025-59009-5)
Supplement: Supplementary file 2 — Description of Additional Supplementary Files [file 41467_2025_59009_MOESM2_ESM.pdf]

Description of Additional Supplementary Files

File name: Supplementary Data 1
Description: Strains and CpaF ortholog accession numbers used for phylogenetic analysis in Fig. 4.

File name: Supplementary Movie 1
Description: Interpolated trajectory between the Com-Apo and Exp-ATP packing units of the hexamer during an ATP binding event. Chains e and f are labelled while the α9 helix and Walker A motif are colored dark red.

File name: Supplementary Movie 2
Description: Interpolated trajectory between the Com-ATP and Exp-ADP1 packing units of the hexamer from an ATP hydrolysis event. Chains f and a are labelled while the α9 helix and Walker A motif are colored dark red.

File name: Supplementary Movie 3
Description: Interpolated trajectory between the Exp-ADP2 and Com-Apo packing units of the hexamer from an ADP release event. Chains a and b are labelled while the α9 helix and Walker A motif are colored dark red.

File name: Supplementary Movie 4
Description: Proposed clockwise rotary mechanism of CpaF catalysis. Iterative interpolated trajectories between the compact and expanded states and back to the compact state, depicting a total of 180° clockwise rotation about the symmetry axis. ATP and ADP are depicted in yellow and cyan, respectively. Each chain is labeled from a to f. The video may be viewed as a loop to visualize a full 360° clockwise rotation.
