# Supplementary material for: Conformational changes in the motor ATPase CpaF facilitate a rotary mechanism of Tad pilus assembly
Source: Nat Commun. 2025 Apr 24;16:3839. doi: 10.1038/s41467-025-59009-5 (PMC12019362; doi:10.1038/s41467-025-59009-5)
Supplement: Supplementary file 8 — Reporting Summary [file 41467_2025_59009_MOESM8_ESM.pdf]

nature portfolio

Corresponding author(s): Lori L. Burrows, Yves V. Brun, P. Lynne Howell

Last updated by author(s): Mar 26, 2025

Reporting Summary

Nature Portfolio wishes to improve the reproducibility of the work that we publish. This form provides structure for consistency and transparency in reporting. For further information on Nature Portfolio policies, see our Editorial Policies and the Editorial Policy Checklist.

Statistics

For all statistical analyses, confirm that the following items are present in the figure legend, table legend, main text, or Methods section.

n/a	Confirmed	
☐	☒	The exact sample size (n) for each experimental group/condition, given as a discrete number and unit of measurement
☐	☒	A statement on whether measurements were taken from distinct samples or whether the same sample was measured repeatedly
☐	☒	The statistical test(s) used AND whether they are one- or two-sided<br>*Only common tests should be described solely by name; describe more complex techniques in the Methods section.*
☒	☐	A description of all covariates tested
☒	☐	A description of any assumptions or corrections, such as tests of normality and adjustment for multiple comparisons
☐	☒	A full description of the statistical parameters including central tendency (e.g. means) or other basic estimates (e.g. regression coefficient) AND variation (e.g. standard deviation) or associated estimates of uncertainty (e.g. confidence intervals)
☐	☒	For null hypothesis testing, the test statistic (e.g. F, t, r) with confidence intervals, effect sizes, degrees of freedom and P value noted<br>*Give P values as exact values whenever suitable.*
☒	☐	For Bayesian analysis, information on the choice of priors and Markov chain Monte Carlo settings
☒	☐	For hierarchical and complex designs, identification of the appropriate level for tests and full reporting of outcomes
☒	☐	Estimates of effect sizes (e.g. Cohen's d, Pearson's r), indicating how they were calculated

Our web collection on statistics for biologists contains articles on many of the points above.

Software and code

Policy information about availability of computer code

Data collection	EPU 2.13, Nikon NIS Elements imaging software, AlphaFold3
Data analysis	cryoSPARC 4.2-4.4, Topaz 0.2.3, Scipion 3.3.0, UCSF ChimeraX 1.7-1.8, Phenix 1.20.1-4487, ISOLDE 1.6, Coot 0.9.8.7, Prism 8, Muscle 5.1, RAxML 8.2.7, iTOL, Alphafold Protein Structure Database, ImageJ

For manuscripts utilizing custom algorithms or software that are central to the research but not yet described in published literature, software must be made available to editors and reviewers. We strongly encourage code deposition in a community repository (e.g. GitHub). See the Nature Portfolio guidelines for submitting code & software for further information.

Data

Policy information about availability of data

All manuscripts must include a data availability statement. This statement should provide the following information, where applicable:

- Accession codes, unique identifiers, or web links for publicly available datasets
- A description of any restrictions on data availability
- For clinical datasets or third party data, please ensure that the statement adheres to our policy

Cryo-EM consensus, locally refined, and composite maps, respectively, have been deposited in the Electron Microscopy Data Bank while the corresponding atomic models have been deposited in the Protein Data Bank under accession numbers: Closed-Apo (EMD-47431, -47432, -47433, 9E24), Compact-Apo (EMD-47434, -47435, -47436, 9E25), Compact-Undersaturated-ATP/ADP (EMD-47437, -47438, -47439, 9E26), Expanded-Undersaturated-ATP/ADP (EMD-47440, -47441, -47442, 9E27), and Expanded-Saturated-ATP (EMD-47444, -47445, -47446, 9E29). CpaF orthologs used to generate the phylogenetic tree in Figure 4 are listed in

Supplementary Table 2. Uniprot accession numbers of proteins used for AlphaFold3 predictions are listed in Supplementary Table 3.

Research involving human participants, their data, or biological material

Policy information about studies with human participants or human data. See also policy information about sex, gender (identity/presentation), and sexual orientation and race, ethnicity and racism.

Reporting on sex and gender	n/a
Reporting on race, ethnicity, or other socially relevant groupings	n/a
Population characteristics	n/a
Recruitment	n/a
Ethics oversight	n/a

Note that full information on the approval of the study protocol must also be provided in the manuscript.

Field-specific reporting

Please select the one below that is the best fit for your research. If you are not sure, read the appropriate sections before making your selection.

[x] Life sciences [ ] Behavioural & social sciences [ ] Ecological, evolutionary & environmental sciences

For a reference copy of the document with all sections, see nature.com/documents/nr-reporting-summary-flat.pdf

Life sciences study design

All studies must disclose on these points even when the disclosure is negative.

Sample size	The number of micrographs and particle images were chosen so that high-resolution structures could be determined. Sample sizes for Tad pili quantification were chosen based on historical data (see Ellison et al Science Advances 2019).
Data exclusions	Particle images that do not correspond to CpaF were excluded during 2D and 3D classifications.
Replication	All in vitro and in vivo assays were performed in three biological replicates, each with three technical replicates.
Randomization	No randomization was used in these experiments. All bacterial strains were grown and manipulated under similar conditions.
Blinding	No blinding was used in these experiments. All bacterial strains were grown and manipulated under similar conditions.

Reporting for specific materials, systems and methods

We require information from authors about some types of materials, experimental systems and methods used in many studies. Here, indicate whether each material, system or method listed is relevant to your study. If you are not sure if a list item applies to your research, read the appropriate section before selecting a response.

Materials & experimental systems

n/a	Involved in the study	
[ ]	[x]	Antibodies
[x]	[ ]	Eukaryotic cell lines
[x]	[ ]	Palaeontology and archaeology
[x]	[ ]	Animals and other organisms
[x]	[ ]	Clinical data
[x]	[ ]	Dual use research of concern
[x]	[ ]	Plants

Methods

n/a	Involved in the study	
[x]	[ ]	ChIP-seq
[x]	[ ]	Flow cytometry
[x]	[ ]	MRI-based neuroimaging

Antibodies

Antibodies used	1:5000 dilution of a polyclonal Caulobacter crescentus NA1000 α-CpaF antibody (Biomatik)<br>1:5000 dilution of a Caulobacter crescentus NA1000 α-GAPDH antibody produced from New Zealand white rabbits (Biomatik)<br>1:5000 dilution of horseradish peroxidase (HRP)-conjugated goat anti-rabbit secondary antibody (Pierce, #1858415)

Validation	Antibodies were used to probe C. crescentus cell lysates for expression of CpaF as demonstrated in Supplementary Figure 12.

Plants

Seed stocks	*Report on the source of all seed stocks or other plant material used. If applicable, state the seed stock centre and catalogue number. If plant specimens were collected from the field, describe the collection location, date and sampling procedures.*
Novel plant genotypes	*Describe the methods by which all novel plant genotypes were produced. This includes those generated by transgenic approaches, gene editing, chemical/radiation-based mutagenesis and hybridization. For transgenic lines, describe the transformation method, the number of independent lines analyzed and the generation upon which experiments were performed. For gene-edited lines, describe the editor used, the endogenous sequence targeted for editing, the targeting guide RNA sequence (if applicable) and how the editor was applied.*
Authentication	*Describe any authentication procedures for each seed stock used or novel genotype generated. Describe any experiments used to assess the effect of a mutation and, where applicable, how potential secondary effects (e.g. second site T-DNA insertions, mosiacism, off-target gene editing) were examined.*
